# Solitary plasmacytoma of the skull: Two case reports

**DOI:** 10.3892/ol.2012.1046

**Published:** 2012-11-26

**Authors:** LUN DONG, XIAN ZHANG, HENGZHU ZHANG, RUIHONG SONG, XUEWEN GU, LIANG HE

**Affiliations:** 1 Departments of Neurosurgery, Clinical Medical College, Yangzhou University, Yangzhou 225001, Jiangsu, P.R. China; 2 Pathology, Clinical Medical College, Yangzhou University, Yangzhou 225001, Jiangsu, P.R. China

**Keywords:** plasmacytoma, skull, diagnosis, therapy

## Abstract

Solitary plasmacytoma of the skull is rare and few cases have been reported in the English literature. Plasmacytoma of the skull has a wide spectrum of pathology, including a quite benign, solitary plasmacytoma (SPC), and an extremely malignant, multiple myeloma (MM) at the two ends of the spectrum. The prognosis for solitary plasmacytoma of the skull appears to be good when it can be diagnosed on strict criteria. The clinical features of solitary plasmacytoma of the skull are complex and not easily identified, resulting in a high misdiagnosis rate. A comprehensive examination and analysis which includes radiological examination, immunoglobulin, biochemistry, test for Bence Jones protein in the urine and bone marrow is needed for correct diagnosis. If the skull lesion is isolated, with accompanying marked swelling in the area and tenderness, plasmacytoma must be considered as a possibility for the cause of solitary skull masses. Two cases of solitary plasmacytoma of the skull lesions were retrospectively reviewed, in which a comprehensive examination was used in order to predict the clinical course of solitary plasmacytoma of the skull. The patients received postoperative radiation and/or chemotherapy. Survival following surgery was longer than 2 years for patient 1, and patient 2 is alive at the 18-month follow-up.

## Introduction

Pathological proliferation of the plasma cell population produces a wide spectrum of disorders, ranging from benign solitary plasmacytoma to malignant multiple myeloma (1). Myeloma is a type of clonal hematopathy. Solitary plasmacytoma of the skull is a rare plasma cell tumor which represents the proliferation of monoclonal plasma cells and produces monoclonal immunoglobulin. Osteolytic skull lesions are commonly observed in routine clinical work. The appearance of a single osteolytic plasmacytoma of the skull without signs of systemic myelomatosis is extremely rare (2,3). The prognosis for solitary plasmacytoma of the cranial vault appears to be good when it is diagnosed on strict criteria, which is based on a radiologically solitary bone lesion, neoplastic plasma cells in the biopsy specimen, <5% plasma cells in bone marrow, <2.0 g/dl monoclonal protein in the serum when present and negative urine test for Bence Jones protein (monoclonal light chain) (4). Hence, making the appropriate diagnosis is critical. The present study describes two cases of skull solitary plasmacytoma, discusses the relevant literature concerning this disease and raises the issue of newer diagnosis and therapy modalities for this disease. The study was approved by the ethics committee of the Clinical Medical College of Yangzhou University, Yangzhou, China and informed consent was obtained from each patient’s family.

## Case reports

### Case 1

A 70-year-old female with a 10-year history of diabetes mellitus first noted a rubbery swelling 4×5 cm in diameter in the parietal region in June 2003. Neurological examination identified a relatively fixed mass with no tenderness and no abnormalities. Computed tomography (CT) showed a large extradural mass with homogeneous enhancement following intravenous administration of contrast material, and bone CT revealed a solitary osteolytic lesion involving the whole layer of the skull. Cranial magnetic resonance imaging (MRI) scan revealed that the occupying lesion in the right frontal and parietal skull was mostly isointense with the brain parenchyma on both T1- and T2-weighted images and was homogeneously enhanced. Laboratory examinations showed a red blood cell count of 3.15×10^12^/l; hemoglobin (HGB), 10^9^ g/l; white blood cell (WBC) count, 4.8×10^9^/l; neutrophils, 49.5%; lymphocytes, 39.5%; which were all within the normal range. A urine test for Bence Jones protein was negative. Renal function showed abnormal levels of urea nitrogen (BUN; 9.38 mmol/l) and creatinine (CRE; 203.6 *μ*mol/l).

The patient underwent a craniectomy under general anesthesia on June 6, 2003. An ∼28-cm horseshoe-shaped incision was made in the right frontal-parietal bone. The tumor extended to the subcutaneous and the subdural space through the dura mater with skull defects ∼4×4 cm. The tumor was an enhancing extracranial mass ∼4×4×1.5 cm. It was purple, soft, had a rich blood supply and was easily separated from the skull. The marginal bone around the tumor was rongeured out to ensure the complete removal of the tumor. The tumor was completely resected, including the marginal bone but without dural lesions, forming a bone window ∼7×8 cm in size. The tumor was ∼5×6×3 cm.

**Figure 1.**
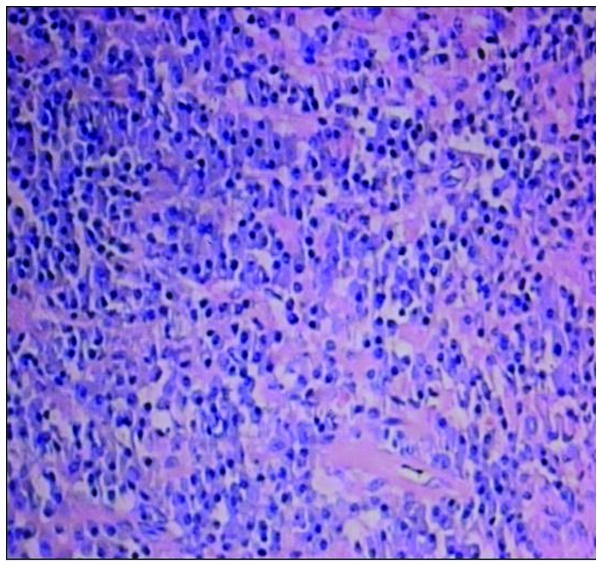
Case 1. Plasma cell tumor (skull). Atypical plasma cells with typical eccentric round nuclei and prominent cytoplasm. (H&E, ×200).

**Figure 2.**
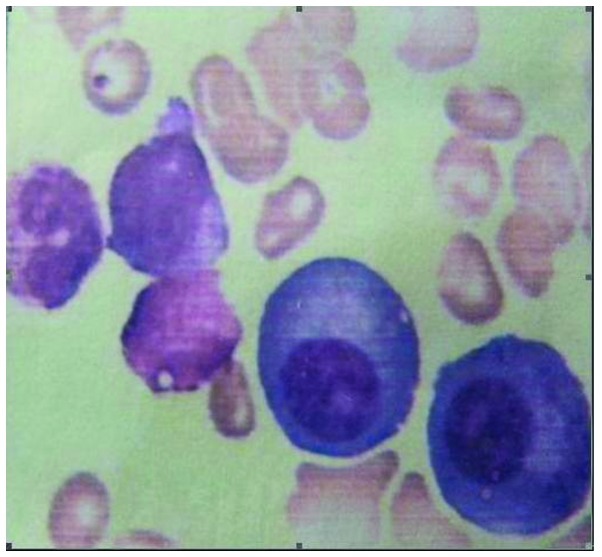
Case 1. Bone marrow examination revealed solitary plasmacytoma (skull).

Pathological diagnosis of the tumor was plasmacytoma (right frontoparietal; Fig. 1). Bone marrow aspiration revealed multiple systemic myelomatosis, and displayed an increasing quantity of plasmacytes after each period of time (Fig. 2). X-ray revealed signs of multiple myeloma in the skull and pelvis.Chemotherapy (melphalan) treatment was administered in the Department of Hematology and started on June 13, 2003. Immunohistochemistry showed embryonal membrane antigen (EMA)(−), GFAP(−) and an erythrocyte sedimentation rate of 120 mm/h. The immunoelectrophoresis of serum proteins revealed that the levels of immunoglobulins (Igs) were: IgG, 4.54 g/l; IgM, 0.17 g/l; IgA 14.9 g/l, β-2 microglobulin, 2.35 mg/l; all were within the normal range. The patient received postoperative chemotherapy. Two years later, CT review was unable to identify the tumor.

### Case 2

**Figure 3.**
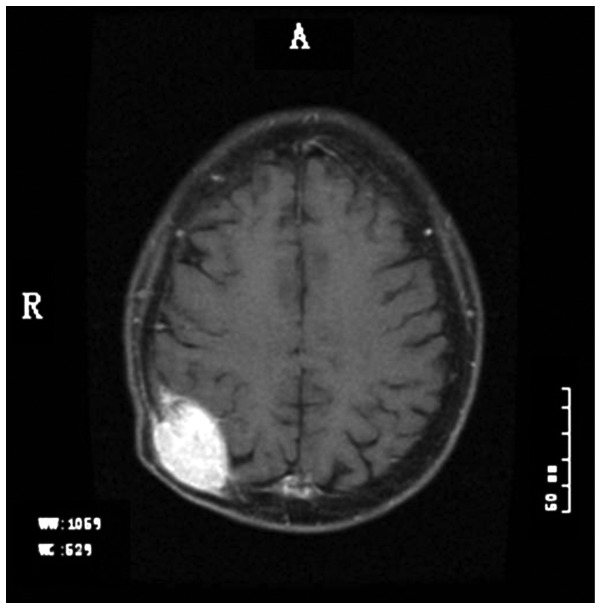
Case 2. Mass destruction and skull defect in the right parietal bone by magnetic resonance imaging.

**Figure 4.**
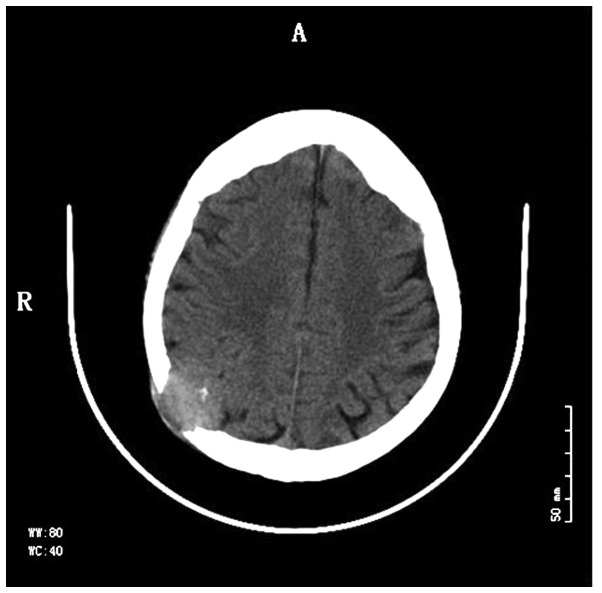
Case 2. Mass destruction and skull defect in the right parietal bone by computed tomography.

A 75-year-old female first noted a rubbery swelling in the right parietal bone for half a month in April 2010. There was a subcutaneous lump ∼3×3 cm in size at the right parietal bone. The border of the mass was clear, smooth and soft, and there was no tenderness. The skull around the lump was defective. The patient had a history of diabetes and renal disease. Cranial CT and MRI scan and enhancement showed the osteolytic defects in the right parietal bone (Figs. 3 and 4). Chest CT scan showed no abnormality. Laboratory examinations revealed a red blood cell count of 2.59×10^12^/l; WBC count, 4.1×10^9^/l; neutrophils, 74.5%; lymphocytes, 19.6%; HGB, 79 g/l; which were all within the normal range. Renal function tests showed renal dysfunction and abnormal levels of BUN (18.36 mmol/l) and CRE (381.6 *μ*mol/l). A test for Bence Jones protein in the urine was weakly positive. The tumor markers carcinoembryonic antigen (CEA; 1.65 ng/ml) and rapid plasma reagin (RPR; negative) were both within the normal range, but the level of serum β-2 microglobulin (5.31 mg/l) was abnormal. Bone marrow aspiration revealed evidence of multiple myeloma. The surgery was abandoned, and the patient was treated with chemotherapy and radiation therapy. After 18 months, CT review was unable to identify the tumor.

## Discussion

Myeloma is a malignant tumor which originates from the reticulocytes of the bone marrow. The tumor cells have the characteristics of an increasing quantity of plasmacytes, so the disease was known as plasma cell myeloma (2). Plasma cell myeloma mostly occurs in the elderly over 40 years of age; the median age of individuals with the disease in the United States was 62 years old (2). Only 2–3% of patients with the disease are younger than 30 years old (3). The two patients described in the present study were over the age of 70, and this is similar to the literature. Bone destruction due to myeloma may occur in any area of the body. Its incidence is as follows: spine, 49%; skull, 35%; pelvis, 34%; ribs, 33%; humerus, 22%; femur, 13%; mandible, 10% (2). Tumors which occur in the skull are called cranial myelomas and are also known as cranial plasma cell tumors. Single tumors are rarely seen in the clinic (3). The preoperative diagnosis of Case 1 was meningioma, the postoperative pathological diagnosis was plasma cell tumor and the bone marrow examination made a definite diagnosis of multiple myeloma. The imaging diagnosis of Case 2 was metastatic tumors, but during the preoperative discussion the anemia and renal dysfunction were found and skull myeloma was suspected. The surgery was abandoned, as the diagnosis from the bone marrow examination was multiple myeloma.

The diagnosis of skull myeloma also needs to be differentiated from eosinophilic granuloma, osteosarcoma and metastatic carcinoma (3).Eosinophilic granuloma is mainly observed in children and young individuals, and most occur singly (85%), which aids the identification of skull myeloma. Eosinophilic granuloma may mostly exhibit bone lesions or a large number of eosinophilic granulomas. The skull is the predilection site, with tumors often located in the frontal, temporal and parietal bones. Tumors have a rich blood supply but clear boundaries from the surrounding tissues. The size of eosinophilic granuloma is usually small, rarely more than 2–3 cm. The edge of the tumor often has a ‘clivus-like’ or ‘bilateral-like’ appearance, and a sequestrum of the ‘button’ type may be observed. Osteosarcoma is a common primary maligant bone tumor which is mostly found in long bones, with few cases in the skull. Osteosarcoma may be divided into osteolytic type, bone and mixed type. The osteolytic type mainly shows bone destruction with a round or irregular shape, with blurred contours. The bone destruction mainly occurs in the outer cranial plate and large extracranial soft tissue is often observed, in which the bone tumor may be found. Osteolytic metastasis is the most common type of metastasis, and the metastases are often multiple and of a range of sizes, with osteolytic bone destruction. The edges of metastases may be blurred and there may be an associated small adjacent soft tissue mass. A small number of osteolytic metastases show single bone destruction with a larger soft tissue mass in which residual bone chips may be observed.

Naganuma *et al* suggested that laboratory examination should include bone marrow examination, serum protein electrophoresis, serum immunoglobulins, blood, urine Bence Jones protein and kidney function (5).

The International Myeloma Working Group proposed new criteria for the diagnosis and classification of myeloma based on routinely available examinations. According to the criteria, symptomatic myeloma requires evidence of an M-protein in the serum and urine, bone marrow plasmacytosis and related end-organ damage (6). The criteria for asymptomatic, or smouldering, myeloma are M-protein levels ≥30 g/l and/or bone marrow clonal cells ≥10%, but no related organ or tissue impairment (ROTI; end-organ damage). Cases with ROTI typically present with increased calcium levels, renal insufficiency, anemia or bone lesions, which are attributed to the proliferation of plasma cells. Symptomatic myeloma requires evidence of ROTI. Solitary plasmacytoma of bone, extramedullary plasmacytoma and multiple solitary plasmacytomas (+/− recurrent) are also defined as distinct entities. The use of these criteria should facilitate the comparison of therapeutic trial data (7). The results of the bone marrow examination confirmed the diagnosis in Case 2.

Prior to 2011, there were only hundreds of cases of solitary plasmacytoma reported in the English literature (8). In cases with no lesions in other parts of the body, the patients have good prognosis following surgical resection and radiotherapy. Chemotherapy is being increasingly used in the treatment of plasma cell myeloma, but radiotherapy is being used less. The prognosis of multiple myeloma is not as good as solitary plasmacytoma (9).

The patient described in Case 1 underwent a frontal-temporal bone craniectomy. During the surgery it is important to control bleeding, and new methods different from the conventional procedure for craniotomy should be selected. If the bone milling cutters or wire sawing are used for craniotomy, it is difficult to stop the bleeding in time and greatly increases the risk of surgery. Since the blood supply of myeloma tumors is mainly taken from the surrounding skull, the process of blocking the blood supply should be performed during surgery on the calvaria. Using the rongeur, while cutting the skull along the edge of tumor up to the normal tissue, discontinuous dural suspension should be carried out. Using the method above, we may effectively reduce blood loss. We should also pay more attention to myeloma in the skull base, since it is more difficult to control bleeding there.

The characteristics of myeloma are complicated. Plasmacytoma of the skull has a wide spectrum of pathology, including a quite benign, solitary plasmacytoma (SPC), and an extremely malignant, multiple myeloma (MM) at the two ends of the spectrum. The clinical features are complex and not easily identified, leading to the high misdiagnosis rate. A comprehensive examination and analysis is needed for correct diagnosis, which includes immunoglobulin, biochemistry, urine Bence Jones protein and bone marrow (10). If the CT scan shows changes in the bone and cartilage, the meta-analysis should be performed to identify the diagnosis.
